# *ALKBH5* gene polymorphisms and Wilms tumor risk in Chinese children: A five‐center case‐control study

**DOI:** 10.1002/jcla.23251

**Published:** 2020-02-24

**Authors:** Rui‐Xi Hua, Jiabin Liu, Wen Fu, Jinhong Zhu, Jiao Zhang, Jiwen Cheng, Suhong Li, Haixia Zhou, Huimin Xia, Jing He, Zhenjian Zhuo

**Affiliations:** ^1^ Department of Pediatric Surgery Guangzhou Institute of Pediatrics Guangdong Provincial Key Laboratory of Research in Structural Birth Defect Disease Guangzhou Women and Children's Medical Center Guangzhou Medical University Guangzhou China; ^2^ Department of Oncology The First Affiliated Hospital of Sun Yat‐sen University Guangzhou China; ^3^ Department of Clinical Laboratory Biobank Harbin Medical University Cancer Hospital Harbin China; ^4^ Department of Pediatric Surgery the First Affiliated Hospital of Zhengzhou University Zhengzhou China; ^5^ Department of Pediatric Surgery the Second Affiliated Hospital of Xi'an Jiaotong University Xi'an China; ^6^ Department of Pathology Children Hospital and Women Health Center of Shanxi Taiyuan China; ^7^ Department of Hematology The Second Affiliated Hospital and Yuying Children's Hospital of Wenzhou Medical University Wenzhou China

**Keywords:** *ALKBH5*, m^6^A, polymorphism, susceptibility, Wilms tumor

## Abstract

**Background:**

Wilms tumor is a frequently diagnosed renal cancer among children with unclear genetic causes. N6‐methyladenosine (m^6^A) modification genes play critical roles in tumorigenesis. However, whether genetic variations of m^6^A modification genes predispose to Wilms tumor remain unclear. *ALKBH5 (AlkB homolog 5)*, a crucial member of m^6^A modification genes, encodes a demethylase that functions to reverse m^6^A RNA methylation.

**Methods:**

Herein, we evaluated the association of single nucleotide polymorphisms (SNPs) in the m^6^A modification gene *ALKBH5* and Wilms tumor susceptibility in a large multi‐center case‐control study. A total of 414 Wilms tumor cases and 1199 healthy controls were genotyped for *ALKBH5* rs1378602 and rs8400 polymorphisms by TaqMan.

**Results:**

No significant association was detected between these two polymorphisms and Wilms tumor risk. Moreover, 1, 2, and 1‐2 protective genotypes (rs1378602 AG/AA or rs8400 GG) did not significantly reduce Wilms tumor risk, compared with risk genotypes only. Stratification analysis revealed a significant relationship between rs1378602 AG/AA genotypes and decreased Wilms tumor risk in children in clinical stage I diseases [adjusted odds ratio (OR) = 0.56, 95% confidence interval (CI) = 0.32‐0.98, *P* = .042]. The presence of 1‐2 protective genotypes was correlated with decreased Wilms tumor risk in subgroups of age > 18 months, when compared to the absence of protective genotypes (adjusted OR = 0.74, 95% CI = 0.56‐0.98, *P* = .035).

**Conclusion:**

Collectively, our results demonstrate that *ALKBH5* SNPs may exert a weak influence on susceptibility to Wilms tumor. This finding increases the understanding of the role of the m^6^A gene in tumorigenesis of Wilms tumor.

## 1 INTRODUCTION

Wilms tumor is a common embryonal kidney that mostly affects children.^1^ It is typically characterized by the disorganized and dysregulated development of a kidney.^2^, ^3^ The prevalence of Wilms tumor is about 7‐10 per million in Western countries, while it is only 3.3 per million in China.^2^, ^4^ Nearly 95% of cases are diagnosed under ten years old, with mean diagnosis age at 43‐48 months.^5^ Survival rates of Wilms tumor in Western countries have reached over 90%,^6^ while the survival rate for relapsed cases is much lower.^7^, ^8^ What is more frustrated, survivors may be subject to chronic severe health conditions.^9^ Strong evidence has been increasingly added in supporting the contribution of genetic variants to Wilms tumor. The *Wilms tumor 1* (*WT1*) gene, mapped to chromosome 11p13, was first identified in 1990 as a tumor suppressor gene in Wilms tumor.^10^ Subsequently, mutations in the *WTX*, *CTNNB1*, and *TP53*, as well as abnormalities of 11p15 methylation have been discovered in Wilms tumor.^11^, ^12^, ^13^, ^14^, ^15^ Apart from these, many other novel gene mutations also have been revealed to be involved in Wilms tumorigenesis.^16^, ^17^ However, all of the above gene mutations affects fewer than 50% of Wilms tumor cases.^18^ Therefore, identifying more variants is indispensable in better understanding the Wilms tumor susceptibility.

N6‐methyladenosine (m^6^A) is the most prevalent and enriched mRNA post‐transcriptional modification.^19^ First discovered in 1974, m^6^A modification is now found to be extensively spread in both prokaryotes and eukaryotes.^20^ The m^6^A modification‐related enzymes are mainly composed of m^6^A methyltransferase (“writers”: METTL3, METTL14, and WTAP), m^6^A demethylases (“erasers”: FTO and ALKBH5), and m^6^A‐binding proteins (“readers”: IGF2BP1 and YTHDF1).^21^, ^22^, ^23^, ^24^ The m^6^A modification plays a critical role in mRNA stability, mRNA translation, and many other important processes.^25^ Dysregulated m^6^A is closely related to various diseases, especially cancers.^26^, ^27^, ^28^ Genetic variants in the m^6^A genes may change the RNA sequences of the target sites or key flanking nucleotide and thereby influence m^6^A modification. The aberrant m^6^A modification level may have impacts on individuals’ cancer susceptibility. The genetic variants in the m^6^A genes are referred to as the m^6^A‐associated SNPs (m^6^A‐SNPs).^29^ The m^6^A modification has become a research hotspot yet studies on the association between m^6^A‐SNPs and cancer risk are very scarce. Therefore, it is urgent to explore the effect of m^6^A‐SNPs on cancer risk, which can provide a new perspective of not only the etiology of cancer but also of the role of m^6^A.

The roles of m^6^A modification gene *ALKBH5* SNPs were recently investigated in the risk of major depressive disorder,^30^ rheumatoid arthritis,^31^ and colorectal cancer.^32^ Till now, no studies have explored the potential relationship of m^6^A modification gene *ALKBH5* SNPs with Wilms tumor risk. In this study, we conducted the first case‐control study of 414 Wilms tumor cases and 1199 controls to yield new insights into the role of m^6^A modification gene *ALKBH5* SNPs in Wilms tumorigenesis.

## 2 METHODS

### 2.1 Study subjects

Wilms tumor cases were enrolled from five hospitals located in Guangzhou, Zhengzhou, Wenzhou, Xi'an, and Taiyuan, respectively.^33^ The current study included a total of 414 cases and 1199 controls of Chinese ancestry, matched on age and gender (Table S1). All Wilms tumor cases were newly diagnosed and pathologically confirmed. No preoperative treatment such as chemotherapy or radiation was performed on the cases before the collection of the blood sample. Healthy controls were recruited in the same period and geographical region. We obtained written informed consent from all participants’ parents or guardians prior to enrolment. Recruitment details and patient characteristics were available in the previously published study.^33^ The study was approved by the Ethics Committee of Guangzhou Women and Children's Medical Center.

### 2.2 Genotyping

Potentially functional SNPs in *ALKBH5* were chosen from the dbSNP database following the criteria described in our previous studies.^34^, ^35^ Briefly, criteria were as follows: (a) located at the two ends of the *ALKBH5* gene (ie, the 5’ near gene, 5’ UTR, 3’ UTR and 3’ near gene); (b) the minor allele frequency (MAF) reported in 1000 Genomes (https://www.ncbi.nlm.nih.gov/variation/tools/1000genomes/) was ≥ 5% for Chinese Han subjects; and (c) affecting transcription factor binding sites (TFBS) activity or the miRNA binding sites activity. As a result, two SNPs (rs1378602 and rs8400) met these criteria. Genomic DNA was extracted from participants’ blood. Samples were genotyped for the rs1378602 and rs8400 SNPs by a custom ABI 7900 HT Sequence Detection System (Applied Biosystems). For sample quality control, we introduced negative control without DNA templates in the genotyping analysis. Moreover, 10% of the samples were re‐genotyped for the SNPs to assess the genotyping error rate. All re‐genotyped SNPs achieved 100% concordance.

### 2.3 Statistical analysis

Characteristics of cases and controls were compared using the chi‐square test or *t* test as appropriate. Compliance of individual SNPs with the Hardy‐Weinberg equilibrium was measured in controls using a chi‐square test. To estimate the association of SNP with Wilms tumor risk, we conducted the unconditional logistic regression analysis. The odds ratios (ORs) and 95% confidence intervals (CIs) were used to determine the association. We also investigated the effect of *ALKBH5* gene SNPs on Wilms tumor susceptibility across strata of age, sex, and clinical stage. False‐positive report probability (FPRP) analysis was further explored to test whether the significant findings were just chance or noteworthy observations. All tests for statistical significance used a two‐sided *P* < .05. Statistical analyses were completed in SAS 9.1 (SAS Institute Inc).

## 3 RESULTS

### 3.1 Association between the *ALKBH5* SNPs and Wilms tumor risk

Patient characteristics were summarized in our previous publication.^33^ The results of the correlation between *ALKBH5* gene polymorphisms and Wilms tumor susceptibility were presented in Table 1. Finally, 413 cases and 1198 controls were successfully genotyped for rs1378602 and rs8400. The genotype frequencies of both SNPs were complied with the Hardy‐Weinberg equilibrium in control subjects (*P* = .488 for rs1378602 and *P* = .963 for rs8400). Neither of these two polymorphisms displayed a significant association with Wilms tumor risk. We then regarded rs1378602 AG/AA or rs8400 GG genotypes as protective genotypes to further explore the combined effects of the two SNPs. However, carriers with 1, 2, and 1‐2 protective genotypes did not have a lower risk in Wilms tumor than those without protective genotype.

**Table 1:** Association between *ALKBH5* gene polymorphisms and Wilms tumor susceptibility

Genotype	Cases (N = 413)	Controls (N = 1198)	*P* a	Crude OR (95% CI)	*P*	Adjusted OR (95% CI)^b^	*P* b
rs1378602 (HWE = 0.488)							
GG	352 (85.23)	991 (82.72)		1.00		1.00	
AG	59 (14.29)	195 (16.28)		0.85 (0.62‐1.17)	.319	0.84 (0.61‐1.15)	.281
AA	2 (0.48)	12 (1.00)		0.47 (0.11‐2.11)	.323	0.46 (0.10‐2.05)	.307
Additive			.188	0.82 (0.62‐1.10)	.188	0.81 (0.61‐1.09)	.160
Dominant	61 (14.77)	207 (17.28)	.238	0.83 (0.61‐1.13)	.238	0.82 (0.60‐1.12)	.205
Recessive	411 (99.52)	1186 (99.00)	.329	0.48 (0.11‐2.16)	.339	0.47 (0.10‐2.11)	.324
rs8400 (HWE = 0.963)							
GG	136 (32.93)	403 (33.64)		1.00		1.00	
AG	205 (49.64)	583 (48.66)		1.04 (0.81‐1.34)	.749	1.04 (0.81‐1.33)	.783
AA	72 (17.43)	212 (17.70)		1.01 (0.72‐1.40)	.970	1.00 (0.72‐1.40)	.986
Additive			.911	1.01 (0.86‐1.19)	.911	1.01 (0.86‐1.18)	.933
Dominant	277 (67.07)	795 (66.36)	.792	1.03 (0.81‐1.31)	.793	1.03 (0.81‐1.30)	.825
Recessive	341 (82.57)	986 (82.30)	.904	0.98 (0.73‐1.32)	.904	0.98 (0.73‐1.32)	.905
Protective genotypes^c^							
0	217 (52.54)	588 (49.08)		1.00		1.00	
1	195 (47.22)	610 (50.92)		0.86 (0.69‐1.08)	.209	0.86 (0.69‐1.08)	.200
2	1 (0.24)	0 (0.00)		/	/	/	/
1‐2	196 (47.46)	610 (50.92)	.225	0.87 (0.70‐1.09)	.225	0.87 (0.69‐1.09)	.216

Abbreviations: CI, confidence interval; HWE, Hardy‐Weinberg equilibrium; OR, odds ratio.

a chi‐square test for genotype distributions between Wilms tumor cases and cancer‐free controls.

b Adjusted for age and gender.

c Protective genotypes were carriers with rs1378602 AG/AA or rs8400 GG genotypes.

### 3.2 Stratification analysis

We further performed a stratified analysis by age, gender and clinical stages (Table 2). The protective effect of rs1378602 AG/AA genotypes was pronounced in the subgroup of children with clinical stage I diseases (adjusted OR = 0.56, 95% CI = 0.32‐0.98, *P* = .042). However, no significant association with Wilms tumor risk was found for the rs8400 in the stratification analysis. In subgroups of age > 18 months, the existence of 1‐2 protective genotypes was associated with 0.74‐fold decreased risk of Wilms tumor, when compared to 0 protective genotypes (adjusted OR = 0.74, 95% CI = 0.56‐0.98, *P* = .035).

**Table 2:** Stratification analysis of *ALKBH5* gene polymorphisms with Wilms tumor susceptibility

Variables	rs1378602 (cases/controls)		AOR (95% CI)^a^	*P* a	rs8400 (cases/controls)		AOR (95% CI)^a^	*P* a	Protective genotypes (cases/controls)		AOR (95% CI)^a^	*P* a
	GG	AG/AA			GG	AG/AA			0	1‐2		
Age, month												
≤18	118/377	25/89	0.90 (0.55‐1.47)	.673	53/147	90/319	0.79 (0.54‐1.17)	.245	66/230	77/236	1.13 (0.77‐1.64)	.535
>18	234/614	36/118	0.78 (0.52‐1.16)	.220	83/256	187/476	1.22 (0.90‐1.65)	.199	151/358	119/374	**0.74 (0.56‐0.98)**	**.035**
Gender												
Females	166/417	28/102	0.69 (0.44‐1.09)	.112	61/172	133/347	1.09 (0.76‐1.55)	.654	105/245	89/274	0.76 (0.54‐1.05)	.099
Males	186/574	33/105	0.96 (0.63‐1.47)	.856	75/231	144/448	0.99 (0.72‐1.37)	.966	112/343	107/336	0.97 (0.71‐1.31)	.837
Clinical stages												
I	122/991	15/207	**0.56 (0.32‐0.98)**	**.042**	52/403	85/795	0.81 (0.56‐1.17)	.256	70/588	67/610	0.92 (0.64‐1.31)	.638
II	93/991	23/207	1.15 (0.71‐1.86)	.576	32/403	84/795	1.31 (0.86‐2.00)	.214	61/588	55/610	0.87 (0.59‐1.27)	.459
III	81/991	13/207	0.79 (0.43‐1.44)	.438	29/403	65/795	1.17 (0.74‐1.84)	.507	52/588	42/610	0.77 (0.51‐1.18)	.229
IV	40/991	9/207	1.09 (0.52‐2.29)	.821	13/403	36/795	1.42 (0.75‐2.72)	.284	28/588	21/610	0.72 (0.40‐1.28)	.262
I + II	215/991	38/207	0.81 (0.56‐1.18)	.279	84/403	169/795	1.00 (0.75‐1.33)	.995	131/588	122/610	0.89 (0.68‐1.17)	.418
III + IV	121/991	22/207	0.89 (0.55‐1.43)	.620	42/403	101/795	1.24 (0.85‐1.82)	.267	80/588	63/610	0.76 (0.53‐1.07)	.115

Abbreviations: AOR, adjusted odds ratio; CI, confidence interval.

The values that are 95% CIs excluded 1 or *P* < .05 are indicated in bold.

a Adjusted for age and gender, omitting the corresponding stratify factor.

### 3.3 False‐positive report probability results

We preset 0.2 as the FPRP threshold. As shown in Table S2, at the prior probability of 0.1, all of the significant findings disappeared. At a prior probability level of 0.25, the decreased Wilms tumor risk remains noteworthy in carriers with protective genotypes 1‐2 for the children > 18‐month subgroup.

## 4 DISCUSSION

This work was motivated by the discovery of m^6^A modification genes as critical cancer regulators and the emerging role of m^6^A gene SNPs in cancer susceptibility. Thus, we proposed a potential contributing role of m^6^A SNPs in Wilms tumor risk. Herein, we attempted to investigate whether *ALKBH5* gene SNPs could link to the risk of Wilms tumor. Our data suggested a weak association between *ALKBH5* gene SNPs and Wilms tumor risk in Chinese children. To date, this is the first report focusing on the association between the *ALKBH5* gene SNPs and Wilms tumor risk.

The m^6^A demethylases include FTO and ALKBH5, both of which belong to the AlkB family.^36^ ALKBH5 was firstly found to have demethylation activity in 2013.^24^ FTO‐mediated m^6^A demethylation generates two intermediates, N6‐hydroxymethyladenosine (hm^6^A) and N6‐formyladenosine (f^6^A), which were finally hydrolyzed into adenine.^37^, ^38^ Unlike FTO, ALKBH5 catalyzes the direct removal of m^6^A without generating an intermediate.^39^ Silencing of *ALKBH5* led to the increase in the total m^6^A levels on RNA as well as the boost of RNAs exportation from the nucleus to the cytoplasm.^24^ Moreover, *ALKBH5* also significantly affects RNA metabolism and the assembly of mRNA processing factors.^24^ *ALKBH5* is critically implicated in the development and progression of several malignancies. Zhang et al^40^ found that expression of *ALKBH5* was upregulated in glioblastoma stem‐like cells (GSCs). *ALKBH5* regulates *FOXM1* gene expression, consequently affecting GSC tumorigenesis. Enhanced *ALKBH5* induced by hypoxia decreases the level of methylated *NANOG* mRNA. The increased NANOG protein levels promote the enrichment of breast cancer stem cell (BCSC) population. Conversely, knockdown of *ALKBH5* impairs tumor formation in vivo by decreasing hypoxia‐induced NANOG expression and BCSC enrichment.^41^ It was also reported that overexpression of *ALKBH5* promotes invasion and metastasis of gastric cancer by demethylating the lncRNA *NEAT1*.^42^ Panneerdoss et al^43^ revealed that *ALKBH5* exerts its pro‐tumorigenic role by regulating m^6^A levels of angiogenesis‐associated and epithelial‐mesenchymal transition transcripts. They provided evidence that collaboration among writers, erasers, and readers regulates cancer growth and progression. Although the significance of the m^6^A gene in cancer is highly appreciated, the study of m^6^A‐SNPs is a nascent field as yet.

*FTO*, as well as its SNPs, were revealed to be strongly associated with various human diseases, mainly obesity, and cancer.^44^, ^45^, ^46^ Unlike *FTO*, information of *ALKBH5* SNPs was still limited. Only until recently has it begun to be realized that m^6^A‐SNPs in the *ALKBH5* account for genetic predisposition to complex traits, such as cancer. Du et al^30^ reported that SNP rs12936694 in the *ALKBH5* gene plays a significant role in conferring to the risk of major depressive disorder in the Chinese Han population. A recent study has shown that 21 SNPs in the *ALKBH5* gene were significantly associated with the risk of rheumatoid arthritis in Asian and European populations.^31^ Most recently, Meng et al performed the first case‐control study regarding m^6^A SNPs and cancer risk. Their study comprised of two stages, discovery stage with 1150 colorectal cancer cases and 1342 controls, and validation stage with 932 colorectal cancer cases and 966 controls. They comprehensively analyzed 240 SNPs in 20 m^6^A modification‐related genes. Among them, only the *SND1* gene rs118049207 contributes to the development of colorectal cancer in the Chinese population. They circumstantiated that rs118049207 change the mRNA of *SND1* gene, and then lead to m^6^A level alteration. SNPs rs2124370, rs8400, rs9899249, rs9913266, and rs2925137 in the *ALKBH5* gene were not associated with colorectal cancer risk.^32^ Given that *FTO*‐SNPs are involved in cancer risk, we have reason to believe that *ALKBH5* gene SNPs exert a similar role. Due to extremely low prevalence, studies specifically in this area of Wilms tumor have not been conducted. Thus, it is of a great necessity to investigate the association between *ALKBH5* gene SNPs and the risk of Wilms tumor. The current clinical analysis provided only a weak impact of *ALKBH5* gene SNPs on susceptibility to Wilms tumor. We speculate the insufficient statistical power caused by the moderate sample size, relative weak effects of single polymorphism, and the influence of other potential pertinent factors may work together to generate such results. To be noted, positive associations were only detected for rs1378602 AG/AA genotypes and 1‐2 protective genotypes under certain subgroups. These data observed in this study are in accordance with the perception of cancer susceptibility, which represents a genetic attribute that modify the possible cancer risk under the influence of environmental conditions or lifestyles. Therefore, significant associations observed here needed to be detected in a larger study with other factors included. Alternatively, these results could be because of chance, which call for larger and validation studies.

The strengths of our study include its good design, multicentric analysis, and relatively large sample size. However, we cannot neglect its accompanied shortcomings. First, although our study was large, the stratified analyses were still limited in power due to the relatively small sample size. The significant findings might be chance observations (FPRP values larger than 0.2 at the prior probability level of 0.1). Therefore, the conclusion obtained here must be viewed as preliminary and needs to be confirmed. Second, all the included participants were Chinese based population. The single population here limits the applicability of the findings to other ethnicities. Last, the current study focuses on only the relationship of m^6^A‐SNPs with cancer risk. The specific mechanisms underlying the effect of the abovementioned m^6^A‐SNPs genotypes on cancer risk should be investigated.

To the best of our knowledge, this is the first large‐scale and multi‐center evaluation of SNPs of key candidate genes involved in the m^6^A pathway and Wilms tumor susceptibility. The observed association should be further validated in another well‐designed analysis with other larger ethnicities.
