# Supplementary material for: ALKBH5 gene polymorphisms and Wilms tumor risk in Chinese children: A five‐center case‐control study
Source: J Clin Lab Anal. 2020 Feb 24;34(6):e23251. doi: 10.1002/jcla.23251 (PMC7307367; doi:10.1002/jcla.23251)
Supplement: Supplementary file 1 [file JCLA-34-e23251-s001.doc]

| **Supplemental Table 1**. Frequency distribution of selected variables in Wilms tumor patients and cancer-free controls | | | | | |
| --- | --- | --- | --- | --- | --- |
| Variables | Cases (N=414) | | Controls (N=1199) | | *P ^a^* |
|  | No. | % | No. | % |  |
| Age range, month | 1-148.63 | | 0.03-156 | | 0.118 |
| Mean ± SD | 31.14 ± 24.27 | | 32.31 ± 26.15 | |  |
| ≤18 | 143 | 34.54 | 466 | 38.87 |  |
| >18 | 271 | 65.46 | 733 | 61.13 |  |
| Gender |  |  |  |  | 0.218 |
| Female | 194 | 46.86 | 520 | 43.37 |  |
| Male | 220 | 53.14 | 679 | 56.63 |  |
| Clinical stages |  |  |  |  |  |
| I | 137 | 33.09 |  |  |  |
| II | 116 | 28.02 |  |  |  |
| III | 94 | 22.71 |  |  |  |
| IV | 49 | 11.84 |  |  |  |
| NA | 18 | 4.35 |  |  |  |
| SD, standard deviation; NA, not available.  ^a^ Two-sided χ^2^ test for distributions between Wilms tumor patients and cancer-free controls. | | | | | |

| **Supplemental Table 2**. False-positive report probability analysis for the significant associations between *ALKBH5* genotypes and Wilms tumor susceptibility | | | | | | | | |
| --- | --- | --- | --- | --- | --- | --- | --- | --- |
| Genotype | Crude OR  (95% CI) | *P* ^a^ | Statistical  power ^b^ | Prior probability | | | | |
|  |  |  |  | 0.25 | 0.1 | 0.01 | 0.001 | 0.0001 |
| rs1378602 AG/AA vs. GG | | | | | | | | |
| Stage I | 0.59 (0.34-1.03) | 0.062 | 0.335 | 0.357 | 0.625 | 0.948 | 0.995 | 0.999 |
| Protective genotypes 1-2 vs. 0 | | | | | | | | |
| >18 month | 0.75 (0.570-0.999) | 0.049 | 0.796 | **0.156** | 0.356 | 0.859 | 0.984 | 0.998 |
| OR, odds ratio; CI, confidence interval.  ^a^ χ^2^ test was used to calculate the genotype frequency distributions.  ^b^ Statistical power was calculated using the number of observations in the subgroup and the OR and *P* values in this table. | | | | | | | | |
